# Integrating physics in deep learning algorithms: a force field as a PyTorch module

**DOI:** 10.1093/bioinformatics/btae160

**Published:** 2024-03-21

**Authors:** Gabriele Orlando, Luis Serrano, Joost Schymkowitz, Frederic Rousseau

**Affiliations:** Switch Laboratory, VIB Center for Brain and Disease Research, VIB, Leuven 3000, Belgium; Switch Laboratory, Department of Cellular and Molecular Medicine, KU Leuven, Leuven 3000, Belgium; Switch Laboratory, VIB Center for AI & Computational Biology, VIB, Leuven 3000, Belgium; Centre for Genomic Regulation (CRG), The Barcelona Institute for Science and Technology, Dr Aiguader 88, Barcelona 08003, Spain; Universitat Pompeu Fabra (UPF), Barcelona, Spain; IC REA, Pg. Lluis Companys 23, Barcelona 08010, Spain; Switch Laboratory, VIB Center for Brain and Disease Research, VIB, Leuven 3000, Belgium; Switch Laboratory, Department of Cellular and Molecular Medicine, KU Leuven, Leuven 3000, Belgium; Switch Laboratory, VIB Center for AI & Computational Biology, VIB, Leuven 3000, Belgium; Switch Laboratory, VIB Center for Brain and Disease Research, VIB, Leuven 3000, Belgium; Switch Laboratory, Department of Cellular and Molecular Medicine, KU Leuven, Leuven 3000, Belgium; Switch Laboratory, VIB Center for AI & Computational Biology, VIB, Leuven 3000, Belgium

## Abstract

**Motivation:**

Deep learning algorithms applied to structural biology often struggle to converge to meaningful solutions when limited data is available, since they are required to learn complex physical rules from examples. State-of-the-art force-fields, however, cannot interface with deep learning algorithms due to their implementation.

**Results:**

We present MadraX, a forcefield implemented as a differentiable PyTorch module, able to interact with deep learning algorithms in an end-to-end fashion.

**Availability and implementation:**

MadraX documentation, together with tutorials and installation guide, is available at madrax.readthedocs.io.

## 1 Introduction

AlphaFold (Jumper *et al.* 2021) started a revolution in computational biology by allowing researchers to access reliable structural information for virtually any natural protein. The general protein folding problem, however, is still far from being solved: while we can predict the conformations of well-studied proteins, inferring the structure of orphan or artificial proteins is still extremely difficult (Orlando *et al.* 2016). Similarly, deep learning (DL) algorithms struggle to converge to meaningful solutions in structural tasks in the absence of abundant experimental data (Zilian and Sotriffer 2013, Montanucci *et al.* 2019), when algorithms have to learn complex physical rules from a limited number of examples, they often result in inaccurate and biased models. This is, for instance, the case of the prediction of peculiar structures such as antibody-antigen structures, for which limited amount of experimentally solved structures are available and which is still an open problem (Abanades *et al.* 2023, Ruffolo and Gray 2022).

The only currently available solution is to limit the number of trainable parameters in the DL model, thereby reducing the risk of overfitting. Unfortunately, this also limits the learning ability of the algorithm.

An alternative solution to this problem might be to define a set of biophysical rules and integrate them directly in the DL algorithm, therefore freeing it from the daunting task of learning physical rules from a few examples. In other words, it might be useful to define a manifold of the solution space of the network, which represents only the physically sound conformations. Such sets of biophysical rules are commonly called “force-fields,” and they are part of tools such as Rosetta (Rohl *et al.* 2004), FoldX (Guerois *et al.* 2002), CHARMM (Bjelkmar *et al.* 2010), or AMBER (Wang *et al.* 2004).

However, traditional force fields are unsuitable for interacting with DL algorithms for two reasons. First, DL algorithms require calculating gradients for the neural network (NN) weights to perform the learning process. This is only possible when the entire DL pipeline is implemented with data-structures that record the gradient of every operation, which is not the case with traditional force-fields. The inclusion of these force fields as such inside a DL algorithm would therefore block the gradient propagation and arrest the training process. In this paper, we refer to the capability of recording the gradient and transferring it to the next step of a DL pipeline as “PyTorch-differentiability” since PyTorch, a very common DL library, provides data structures to handle gradients. Some advancement has been performed in the field of PyTorch-differentiable force fields in the last years, but they are focused on molecular dynamics simulation applications (Doerr *et al.* 2021, Schoenholz and Cubuk 2020, Wang *et al.* 2023). Such force fields are not suitable to describe the energy of a single protein conformation because they lack the entropic terms. Second, DL training is often very computationally intensive; a force-field, therefore, needs to be as efficient as possible both in energy and in gradient calculations to be used in DL settings. This second requirement can be achieved by implementing every physical calculation as operations between tensors, for which functions, highly optimised at the hardware level, are available (i.e. CUDA).

In this paper we present MadraX, a tool which fulfils both requirements. It is a knowledge-based force field implemented as a PyTorch (Paszke *et al.* 2017) module. We used the FoldX energy function (Guerois *et al.* 2002), which is a well-established force field for which plenty of validations are available (Heselpoth *et al.* 2015, Buß *et al.* 2018), and found a way of transforming its parameters, equations and expressions to make it completely PyTorch-differentiable via the autograd function of PyTorch. Only a few of the terms, for computational and calculation efficiency reasons, were partially modified or integrated (see Methods). MadraX can thus be embedded within any PyTorch NN, like a standard NN layer, maintaining all the properties typical of PyTorch modules.

The online documentation, available at madrax.readthedocs.io, provides tutorials to introduce the user to MadraX.

## 2 Methods

MadraX estimates the energies that stabilise a biological molecule or complex. The energies are defined by hard coding the biophysical formulas in tensorial form and, therefore, no machine learning is involved. MadraX distinguishes 11 classes of interaction/stabilisation energies. Seven of these classes have been taken from the FoldX force field (Schymkowitz *et al.* 2005) and re-implemented in an efficient way in tensorial and PyTorch-differentiable form. In the following paragraphs we are describing the energies taken into consideration by MadraX. All the energies calculated are then sum together to estimate the energy of the protein or complex.

### 2.1 Hydrogen bonds

Hydrogen bonds are one of the most important energies that stabilize a protein conformation, being crucial in the formation of secondary structure elements such as alpha helices and beta sheets. They occur when an atom shares an hydrogen with an acceptor of another residue, which in turn presents a free electron orbital. Madrax defines the energy of hydrogen bonds based on (1) the distance between the hydrogen and the accepting free electron orbital, and (2) the variation of the geometry of the bond in respect to the optimal one. For more information about the implementation of the hydrogen bond energy in MadraX see the the FoldX paper (Schymkowitz *et al.* 2005).

### 2.2 Electrostatic interactions

Electrostatic interactions occur when two charged atoms are close to each other and the resulting energy can be either stabilizing, if the charges have opposite sign, or destabilizing, if they have the same sign. Electrostatic energy contribution is calculated, like in FoldX (Guerois *et al.* 2002), with the Coulomb’s equation, where the interaction between atom *i* and *j* is defined as

(1) $$\begin{array}{c} E_{ij}=\frac{332q_{i}q_{j}}{\varepsilon d_{ij}}e^{-d_{ij}K} \end{array}$$

Where $q_{i}$ and $q_{j}$ are the charges of atom *i* and *j*, respectively, ε is the dielectric constant, $d_{i}j$ is the distance (in Å) between the two atoms and *K* is Debye–Huckel parameter, calculated as

(2) $$\begin{array}{c} K=\sqrt{\frac{8\pi e^{2}NI}{1000kT}} \end{array}$$

where *N* is the Avogadro’s number, *k* the Boltzmann’s constant, *T* the temperature (in K) and *I* the ionic strength of the solvent (in M). The final electrostatic energy is expressed in kCal/mol. For more information about the implementation of the electrostatics in Madrax refer to online documentation.

### 2.3 Disulfide bonds

Disulfide bonds are covalent bonds and are one the strongest interactions found in proteins. They occur when the side chains of two cysteine residues get oxidized, creating a bond between their two sulfur atoms. In Madrax the energy of a disulfide bond is calculated as in FoldX, in which it is based on the distance between the two sulfur atoms and the geometry of interaction between them. For more information about the implementation of the disulfide bonds in Madrax refer FoldX paper (Guerois *et al.* 2002).

### 2.4 Polar and hydrophobic solvation

Solvation represents the effect of the solvent on the energy of the protein. Exposed hydrophilic residues can interact with the solvent, stabilizing the folded protein conformation. On the other hand, exposed hydrophobic residues can promote unfolding and therefore provide a positive contribution to the conformational energy. Madrax estimates solvation in the same way as in FoldX, therefore starting from the free energy required to transfer each amino acid type and other model compounds from water to ethanol, dioxane and n-octanol. A deeper discussion on how to estimate residue-specific free energy transfer and how to connect it to hydrophilic and hydrophobic solvation coefficients can be found in Guerois et al. (2002). These residue-type-specific coefficients are then considered together with the solvent exposure of every residue to calculate the final hydrophobic and hydrophilic solvation energies.

### 2.5 Van der waals energies

Van der Waals energies are implemented are calculated in a conceptually similar way to solvation. In this case, the per-residue free energy coefficients are calculated based on the energy required to transfer every amino acid type from vapour to water. The coefficients are then considered together with the solvent accessibility of each atom. A deeper discussion about the way in which residue-specific coefficients for Van der Waals energy have been calculated refer to Guerois *et al.* (2002).

### 2.6 Clashes

Clashes occur when two atoms are too close, and their Van der Waals spaces partially overlap. Clashes should almost never be present in high-quality protein models, and atom overpacking is expected to be detrimental to protein stability. This energy is calculated in a different way with respect to FoldX since the original function was intrinsically non differentiable. MadraX determines clashes and their magnitude based on the minimum distance between specific pairs of atoms observed in high-resolution crystal structures and their atomic radii. To do so, we first downloaded 5000 high-resolution (<1.5A) x-ray protein structures with length between 100 and 500 amino acids from the PDB and for every atom pair within 5A from each other, we collected the difference between their distance and the sum of their radius. We removed from the calculations all the pairs connected by a covalent bond (excluding disulfide bonds) or that were part of a rigid geometrical structure, such as the atoms belonging to the same aromatic ring or the carbonyl and carbon delta of prolines. We also excluded from the calculation atom pairs that were part of the backbone of the same residue. The remaining pairs of atoms were then grouped in six different groups: (1) pairs belonging to the same residue but not directly connected with a covalent bond, (2) hydrogen bonds, (3) disulfide bonds, (4) atoms of the backbone of consecutive residues, (5) donor-acceptor pairs not forming a hydrogen bond, and (6) all the rest. For each group, we calculated the 10th percentile of the distances minus the sum-of-radii difference, which we call correction (see also the formula below). When the clash energy between two atoms is evaluated, we assign an exponentially growing energetic penalty to all the pairs (*i, j*) associated with a distance that is shorter than the value of its group, using the following formula:

(3) $$\begin{array}{c} \mathrm{Clas}h_{i,j}=w*e^{10*(R_{i}+R_{j}-d_{ij}-t_{g})} \end{array}$$

where *w* is a scaling factor, $R_{i}$ and $R_{j}$ are the radii of atoms *i* and *j*, respectively, $d_{ij}$ is the distance between atom *i* and atom *j* and $t_{g}$ is the correction for the group to which the atom pair *i*, *j* belongs. We decided to use only 5000 structures to limit the amount of computational power required to handle them since we did not observe any significant change in the estimated percentiles for larger number of proteins.

### 2.7 Backbone entropy

Backbone entropy is also calculated in a different way with respect to the original FoldX code. We calculate it as the log-probability of a residue having specific backbone torsion angles. The distribution of angles was estimated from the high resolution crystal structures already used for the estimation clashes, using a PyTorch-differentiable kernel density estimation (KDE) method based on masked autoregressive flow (Papamakarios *et al.* 2017). For every residue type, we define two distributions: one (with 2 dimensions) for the Phi/Psi angles and one for the Omega angle. The latter has two very narrow Gaussian distributions around −180 and 180 degrees. The final backbone entropy is the sum of these two values. In mathematical terms,

(4) $$\begin{array}{c} \mathrm{Entrop}y_{i}=-w_{1}ln(KDE_{o}(\Omega ))-w_{2}ln(KDE_{t}(\phi ,\psi )) \end{array}$$

where $w_{1}$ and $w_{2}$ are two scaling factors, $KDE_{o}$ and $KDE_{t}$ are the two mentioned probability distributions and ϕ,ψ and ω are the backbone torsion angles.

### 2.8 Side chain entropy

Side chain entropy is connected to the degrees of freedom of the side chain and, therefore, to its capability of moving. Madrax calculates it in the same way as FoldX, where an amino acid-specific entropy value, calculated as described in Guerois *et al.* (2002), is scaled by the solvent accessibility of the side chain. In general, this scaling causes exposed residues to pay a weaker entropy penalty. However, the presence of hydrogen bonds, strong electrostatic interactions or disulfide bonds that limit the degrees of freedom of the side chain, make the residue pay the full entropy cost. For a more detailed description of the implementation of the side chains refer to the FoldX article (Guerois *et al.* 2002).

### 2.9 Peptide bond violation

Peptide bonds are covalent interactions between the carbonyl and amino groups of two consecutive residues. Although the geometry of peptide bonds is strongly constrained by atom hybridisation and optimal binding distances, there can be small variations around the optimal values without disrupting protein stability. In order to allow such variability, MadraX gives a penalty to stability based on the deviation from the optimal geometrical values. Specifically, we consider two angles, the CA-N -Cp and CAp-Cp-N, where p indicates an atom of the previous residue, and the distance between the nitrogen and the carbon forming the peptide bond. For each of these values, we built a Gaussian distribution, based on the values observed in the dataset of aforementioned high resolution crystal structures. The final penalty V is then defined as

(5) $$\begin{array}{c} V=w\sum_{i}-ln(G_{i}(X_{i})) \end{array}$$

Where w is a scaling factor, i is the estimator under examination (one of the two angles or the bond length), $G_{i}$ is the Gaussian distribution for the estimator *i* and $X_{i}$ is the value of the estimator *i* for that specific peptide bond. This energy was not included in the original FoldX code.

### 2.10 Side-chain conformation violation penalty

We define the side-chain conformation of a residue by its χ torsion angles. Amino acid side-chains have up to 5 χ torsion angles. This means that, assuming a fixed covalent bond geometry and length in the side chain, every possible conformation can be defined in a space with up to five dimensions, each of which represents a torsion angle. In reality, not all the conformations are physically possible and a large portion of them never or very rarely occur in proteins. To account for this effect, we introduced a side-chain conformation penalty that is calculated using the same KDE approach we used for calculating the backbone entropy, but this time the axis represents the χ angles of the amino acid under scrutiny. The probability distribution estimation has been performed using the same dataset of high-resolution crystal structures described in the previous sections. The final penalty for a specific residue is given by the negative logarithmic probability provided by the kernel. In mathematical terms,

(6) $$\begin{array}{c} \mathrm{SCviolatio}n_{i}(\chi )=-w_{i}ln(\mathrm{KDE}(\chi )) \end{array}$$

Where $w_{i}$ is a scaling factor for residue *i*, KDE is the probability distribution mentioned above for each *chi* torsion angle of the side chain. This energy was not included in the original FoldX code.

Every energy is associated with a scaling factor. The energies needed to be rescaled to take into account the changes with respect to the original FoldX energy function. We assigned them with a grid search maximizing the correlation between FoldX and MadraX ΔΔG of the dataset presented in Pucci *et al.* (2018). For an estimation of the execution times of MadraX refer to Supplementary Fig. S4

## 3. Results

### 3.1 Madrax allows the application of general gradient-based optimizers to proteins

PyTorch is widely recognized as a prominent framework for developing neural networks, predominantly utilizing its auto-differentiation algorithms to compute gradients with respect to neural network weights.

The versatility of auto-differentiation algorithms extends beyond gradients solely associated with neural network weights, enabling the calculation of derivatives for any arbitrary variable.

MadraX, being differentiable in PyTorch, mimics the behavior of a conventional parameter-free neural network layer. Consequently, we can exploit the back-propagation algorithm already implemented in PyTorch to compute gradients of the energy provided by MadraX with respect to other variables. These gradients can be effectively utilized within gradient descent optimization algorithms, which have been developed by leading entities like Facebook and Google for generic purposes unrelated to biology. Importantly, PyTorch offers highly stable and efficient implementations of these optimization algorithms.

By supplying the atom coordinates (*X*) of a protein (*P*) as input to MadraX, we obtain an energy value (*E*). Calculating the gradient δE/δX allows us to determine how the energy (*E*) varies based on the atom positions. We can utilize one of the aforementioned generic optimizers available in PyTorch to relax protein structures, minimizing the energy (*E*) by iteratively adjusting the atom coordinates (*X*) in a manner dictated by the gradient.

Alternatively, we can also differentiate *E* with respect to the torsion angles, thereby maintaining the geometry of covalent bonds within the protein. Simply put, this method maintains the FoldX energy function, reformulating the way in which we find the best conformation as a simple and generic gradient-based optimization problem, a well-known and studied computational task for which plenty of algorithms are available.

Both these optimization procedures are purely gradient-based and do therefore not require any protein backbone fragments or side chain rotamer databases as support. Moreover, this application only requires a couple lines of code that are provided as a tutorial in the MadraX documentation.

To confirm that our protein stability prediction method performs similarly to FoldX, even when we make small changes in energy calculations, we used our approach to predict how mutations affect protein stability. Specifically, we employed MadraX to predict the impact of 342 mutations from the dataset mentioned in reference Pucci *et al.* (2018). The resulting correlation coefficient between the predicted and experimental changes in ΔΔG is 0.49, which is in line with FoldX’s performance. More information about this sanity check is reported in Supplementary Section 2.2.

### 3.2 MadraX constrains the searching space of a network

When tackling structural bioinformatics problems, the generation of a structure often serves as an intermediate step in a larger task. This is the case, for example, of the prediction of the effect of mutations on protein stability, where the accurate prediction of a ΔΔG heavily relies on the conformation mutant structure. A typical pipeline to address these problems is therefore to build a model to predict the conformation of the mutant and then, from it, predict its effect on stability.

If we want to address the problem with a NN in an end-to-end way, where the prediction of the mutant conformation is trained together with the ΔΔG prediction, we will therefore need to build a network made of two parts: the first one will generate the conformation of the mutant, while the second one will take the conformation as input and output the stability change.

However, if the NN lacks appropriate structural constraints, it may generate physically unrealistic structures. Although this issue might not immediately impact the network’s performance during computational validation, it can lead to the network learning incorrect rules, potentially influenced by biases present in the dataset. Evaluating the quality of these learned rules poses a challenge, given that neural networks are inherently opaque black boxes. This challenge becomes even more pronounced when working with limited data, which increases the risk of overfitting.

In this context, MadraX offers a promising solution by interacting with neural networks during the training process. It can effectively be used to penalize the generation of physically unstable conformations, constraining the space of possible protein structures the network have to deal with. Remarkably, MadraX achieves this without requiring additional parameters specifically dedicated to learning the intricate physical rules that govern protein conformation. As a result, it becomes a valuable tool for addressing problems with limited data availability, where traditional learning approaches struggle to capture the complexities of protein structures.

To simulate a problem in this domain, we constructed a network to predict the stability of a specific protein (Human Lysozyme, PDB ID 1LZ1) after a mutation. We chose this protein due to its extensive study and availability of stability experiments, including 61 annotated point mutations with corresponding ΔΔG values from the dataset described in Pucci *et al.* (2018).

For what concerns the network, we used a publicly available architecture used to predict the effect of mutations in antibodies interfaces (Shan *et al.* 2022). The usage of a publicly available architecture allows us to start from pretrained models in a transfer learning way and therefore improving convergence in early stages of training, reducing the required computational resources. The network architecture presented in Shan *et al.* (2022), however, requires the structure of the mutant protein as input. We turned this architecture in an end-to-end approach by implementing a layer which encodes for a rotation of the χ torsion angles of the side chain of the mutant residue (see Section 2 for more details). This simple change allows our network to perform a prediction of ΔΔG starting from the wild type structure and calculating the conformation of the mutant residue as an intermediate step.

We trained two versions of the network: one solely with the ΔΔG data and another incorporating a Madrax-based loss function to impose physical constraints and ensure generation of structurally realistic outputs. Apart from the additional loss function, the two networks are identical. Both networks underwent 20 epochs of training on the available data and were validated with a 3-fold cross-validation.

The two networks do not differ significantly in the performances achieved in ΔΔG prediction (pearson’s CC in between 0.42 and 0.44). When looking to the structures generated for the mutant proteins, however, it is evident that the ones generated with the constrained network respect more the physical rules, while the ones obtained with the unconstrained network often show evident violations. In general, the cross validation highlighted that the constrained network learns to generate structures that, on average, have an estimated MadraX energy that is 7.2 Kcal/mol lower. Supplementary Fig. S3 shows three examples of mutants generated with the constrained and unconstrained network respectively. While this is an expected behavior since the network has been trained using also MadraX energy as a loss function, it highlights the application of a manifold to the output space of the network.

The presented pipeline, as the other examples, should be considered as a proof of concept and not as a predictor. Building such a tool would indeed require the development of proper architectures, which are outside the scope of this article.

It is important to note that the constraint is applied only during training. It does not directly interact with any part of the network or provide energy information as input for any layer. Instead, MadraX influences the network by constraining the search space, ensuring the generation of physically valid structures. Once the network has been trained, MadraX can therefore be neglected without influencing the output of the network during the prediction phase. More information about the network can be found in Supplementary Section 2.2.

## Supplementary Material

btae160_Supplementary_Data
