# Supplementary material for: Integrating physics in deep learning algorithms: a force field as a PyTorch module
Source: Bioinformatics. 2024 Mar 21;40(4):btae160. doi: 10.1093/bioinformatics/btae160 (PMC11007235; doi:10.1093/bioinformatics/btae160)
Supplement: btae160_Supplementary_Data [file btae160_supplementary_data.pdf]

Integrating physics in deep learning algorithms: A force field as a PyTorch module

Orlando et al.

1 Supplementary Figures

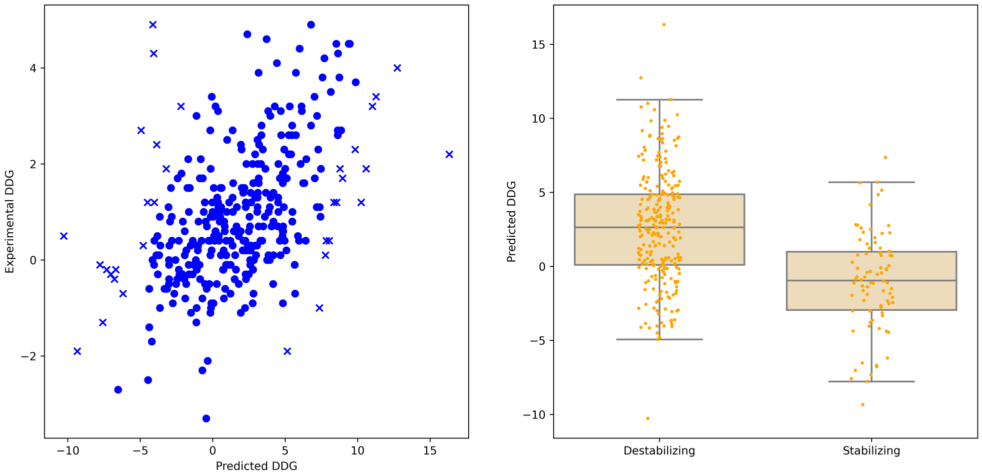

Supplementary Figure 1: **Performance of the protein stability prediction task.** *Left*, Plot of the predicted $\Delta\Delta G$ (horizontal axis) and the experimental ΔΔG(vertical axis). The Pearson's correlation coefficient is 0.49. The 10% of the data points in the validation data set that deviates the most from this correlation is marked by x. Right, box plots of the scores assigned to mutations that destabilize and stabilize the protein folding. The Pearson's correlation coefficient of the original FoldX predictions with the experimental data is 0.5.

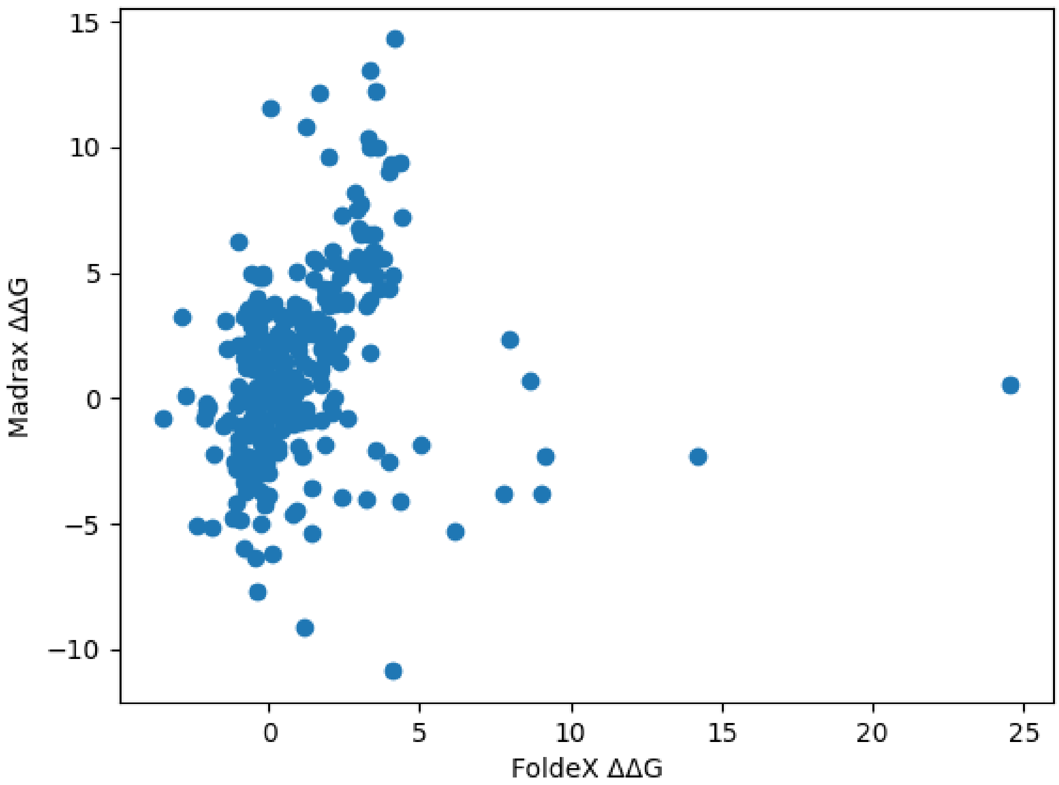

Supplementary Figure 2: Relation between MadraX and FoldX predicted $\Delta\Delta G$. The plot shows the difference between the predicted $\Delta\Delta Gs$ of FoldX and MadraX on the dataset presented in [1]. The $\Delta\Delta G$ of MadraX is calculated after a relaxation step, as described in section a 2.1 of the main article. The $\Delta\Delta G$ of FoldX is calculated using the buildModel command.

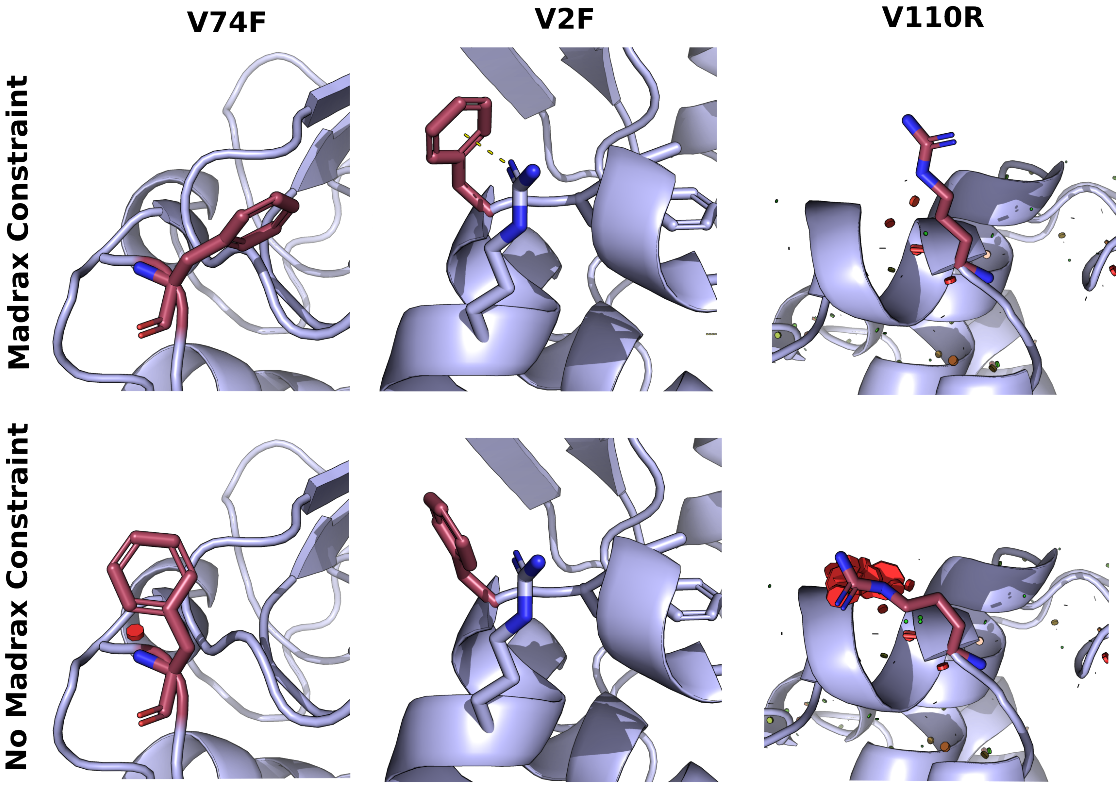

Supplementary Figure 3: **Constraining the searching space of a neural network with Madrax**. In this figure, we present an exploration of how Madrax can effectively constrain the search space of a network, leading to the learning of solutions that align with physical principles. Here we show three examples of mutations in human lysozyme (PDB ID 1LZ1), where the mutated residue is highlighted in red. The top row showcases structures obtained when incorporating Madrax as a constraint, while the second row illustrates structures generated using the same network architecture but without any physical constraints. Notably, in the first mutation, the Madrax constraint guides the network to circumvent a self-clash scenario, represented by the red circle, involving the phenylalanine side chain ring and its own backbone. In the second mutation, the network trained with Madrax positions the side chain in a pose that enables the formation of a cation-pi interaction with an arginine residue. Finally, the constrained network demonstrates its ability to provide a solution that avoids a significant clash with the neighboring alpha helix, an issue observed in the unconstrained network.

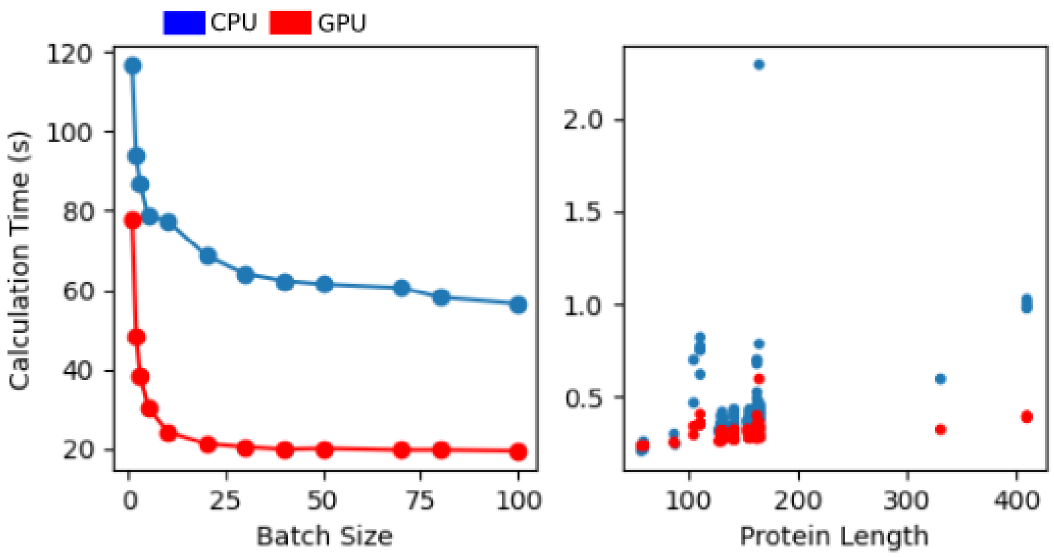

Supplementary Figure 4: Execution time of Madrax: The left panel shows the execution time of MadraX as a function of the batch size. As for most of the neural network layers, MadraX benefits from batched calculations since it allows a more efficient resources utilization. The right panel shows the execution time of MadraX as a function of the protein length. The blue data shows execution on CPU (Intel i9-12900H) and the red ones on GPU (Nvidia GeForce RTX 3080). Both tests have been performed on the dataset described in [1].

2 Discussion of performance and validation

2.1 Protein structure relaxation

To confirm that MadraX maintains the properties and predictive capabilities of a standard force field, we estimated its power to predict the effect of mutations on the stability of proteins. For this purpose, we downloaded a dataset of 342 mutations available on Protherm [2], downloaded from [1], for which a validation using a large number of methods is available [1] (see [1] for the list of mutations taken into consideration), along with the respective wild-type structures.

We implemented the mutations in the respective wild-type structures using MadraX utility function *mutate.mutatingEngine.mutate*. We then defined a NxCxRxMx5 tensor, where N is the number of protein structures, C is the maximum number of chains, R is the maximum number of residues, and M is the maximum number of mutations per protein. This tensor represents the rotation applied to the χ angles of every residue. We used

the MadraX utility function *mutate.rotator.RotateStruct* to implement the rotation. In this function, the mutant and the wild-type protein share all other amino acids with the exception of the mutated ones, and therefore most amino acids are rotated the same way. This gives a huge boost to computational speed because of decreased memory usage and because it reduces the variability in estimation caused by stochastic optimisers. We then used a PyTorch optimizer (Adam) with a learning rate of 10^{-2} for 50 epochs to find the set of rotations that minimize the energy of both the mutant- and the wild-type structures to ensure that both are in a conformation associated with an energetic minimum.

To estimate the effect of mutations, we simply took the difference between the energy of mutant and wild-type proteins. The correlation between the predicted ΔΔG and the experimental one is 0.49 , which is in line with the bests force field-based stability predictors. Supplementary Figure 2 shows the scatter plot between the predicted and experimental ΔΔG. Despite the excellent performance of MadraX this is just an example of the usefulness of MadraX's PyTorch-differentiability, and MadraX should not be considered a predictive tool.

Although the development of a stability prediction tool is outside the scope of this paper, it is technically possible to build a fast and easy-to-use stability predictor with MadraX. Specifically, we think such a tool would primarily benefit from an ad hoc policy to focus on the optimization of the residues that are likely to have a different conformation in the mutant. Such a policy, currently absent in this example, would reduce the number of dimensions the optimizer has to work in, along with the number of differentiation operations that would be required. Protein stability predictors, such as Rosetta or FoldX, never optimize the entire protein because rotating the side chain of every single residue would require such a large amount of rotations that would negatively affect the ability of the optimizer to find the absolute minimum of the energy function. For this reason, limiting the number of rotations the optimizer has to deal with would most probably improve both the speed and the performance of the predictor.

2.2 Madrax constrains the searching space of a network

In this paragraph, we explored the utilization of MadraX to constrain the search space of a neural network during training. This approach penalizes the generation of protein conformations that violate biophysical rules and ensures accurate conformation generation as an intermediate step in relevant tasks.

To illustrate this usage, we constructed a neural network designed to estimate the change in stability ($\Delta\Delta G$) resulting from a protein mutation. The network architecture was based on the work of Shan et al. [3], originally developed for predicting affinity changes in antibodies. We modified the architecture to incorporate a mutation step within the network itself.

Here is an overview of the pipeline: First, the mutation was applied to the wild type protein structure using the MadraX *madrax.mutate* module, without relaxation, which means it does not guarantee adherence to physical constraints. Next, we calculated embedding tensors for the wild type and mutant structures using Shan et al.'s model. These embeddings represented the conformation of the mutating residue, taking neighboring residues into account, and consist in a tensor of shape (128x128).

The embedding of the mutant was fed into a self-attention module consisting of three submodules: an attention module, a prediction module, and a final module. The attention module used a linear layer to transform the embedding into a tensor of shape (128).

Softmax was then applied to ensure the attention weights summed to 1. The prediction module, also a linear layer, mapped the embedding into a tensor of shape (128x50) without applying any activation function. The attention weights were multiplied with the prediction to obtain a tensor of shape (128x50), which was then summed over the first dimension to yield a tensor of shape (50). This tensor served as input for the final module, a two-layer feed-forward network with 50 hidden neurons, a hyperbolic tangent (tanh) activation function, and 5 output neurons.
To obtain the rotation angles for the mutant residue's torsion angles, the final module's output, bounded between -1 and 1, was multiplied by π. These angles were used to rotate the side chain of the mutant residue using the "madrax.mutate.rotator.RotateStruct" module. Thus, we obtained both wild type and mutant structures in an end-to-end fashion, enabling the use of Shan's architecture to calculate the $\Delta\Delta G$.
The network was trained for 20 epochs using an Adam optimizer with a learning rate of 0.0001. We used pretrained Shan's architecture weights to reduce the computational resources to make the network converge. Two training variants were employed: one using mean squared error (MSE) loss on the predicted and experimental $\Delta\Delta G$, and the other incorporating the energy of the predicted mutant structure calculated with MadraX together with the MSE.

Supplementary references

[1] Pucci, F., Bernaerts, K. V., Kwasigroch, J. M. & Rooman, M. Quantification of biases in predictions of protein stability changes upon mutations. *Bioinformatics* **34**, 3659–3665 (2018).

[2] Gromiha, M. M. *et al.* Protherm: thermodynamic database for proteins and mutants. *Nucleic acids research* **27**, 286–288 (1999).

[3] Shan, S. *et al.* Deep learning guided optimization of human antibody against sars-cov-2 variants with broad neutralization. *Proceedings of the National Academy of Sciences* **119**, e2122954119 (2022).
